# Modelling the Mechanical Attributes (Roughness, Strength, and Hardness) of Al-alloy A356 during Sand Casting

**DOI:** 10.3390/ma13030598

**Published:** 2020-01-28

**Authors:** Kashif Ishfaq, Muhammad Asad Ali, Naveed Ahmad, Sadaf Zahoor, Abdulrahman M. Al-Ahmari, Faisal Hafeez

**Affiliations:** 1 Department of Industrial and Manufacturing Engineering, University of Engineering & Technology, Lahore 54890, Pakistan; 2 Department of Industrial Engineering, University of Engineering and Technology, Taxila 47080, Pakistan; 3 Industrial Engineering Department, College of Engineering and Architecture, Al-yamamah University, Riyadh 11421, Saudi Arabia; 4 Industrial Engineering Department, College of Engineering, King Saud University, Riyadh 11421, Saudi Arabia

**Keywords:** A356-alloy, response surface methodology (RSM), scanning electron microscopic analysis, pouring velocity, moisture content, pouring temperature, hardness, roughness, strength

## Abstract

Sand-casting is a well established primary process for manufacturing various parts of A356 alloy. However, the quality of the casting is adversely affected by the change in the magnitude of the control variables. For instance, a larger magnitude of pouring velocity induces a drop effect and a lower velocity increases the likelihood of cold-shut and mis-run types of defects. Similarly, a high pouring temperature causes the formation of hot tears, whereas a low temperature is a source of premature solidification. Likewise, a higher moisture content yields microcracks (due to gas shrinkages) in the casting and a lower moisture content results in the poor strength of the mold. Therefore, the appropriate selection of control variables is essential to ensure quality manufactured products. The empirical relations could provide valuable guidance in this regard. Additionally, although the casting process was optimized for A356 alloy, it was mostly done for a single response. Therefore, this paper aimed to formulate empirical relations for the contradictory responses, i.e., hardness, ultimate tensile strength and surface roughness, using the response surface methodology. The experimental results were comprehensively analyzed using statistical and scanning electron microscopic analyses. Optimized parameters were proposed and validated to achieve castings with high hardness (84.5 HB) and strength (153.5 MPa) with minimum roughness (5.8 µm).

## 1. Introduction

Aluminum and its alloys are widely used in automobiles, aerospace and many other manufacturing facilities due to their better strength-to-weight ratio along with better corrosion resistance [1,2,3]. Sand casting is employed as the primary casting process by industries for casting of aluminum alloys. This process is widely used to produce different sizes and shapes of metal products because it offers ease in manufacturing at a low cost. Therefore, this process has found extensive applications in automobile, aircraft, households and many other manufacturing sectors [4,5]. Although the use of sand-casting process is beneficial, the conventional way of making sand molds is time-consuming with a low production rate. Furthermore, the traditional method of sand casting yields poor mechanical and surface properties because of the many existing inherent porosities that are likely to promote defects in casting. It is pertinent to mention that the casted product quality is mainly governed by the quality of the mold. Therefore, it is essential to enhance the quality of the mold to produce accurate products the first time. Nowadays, competitive markets’ fulfillment of customers’ desired quality in adequate lead-time is mandatory for the survival of the industry. The issues pertaining to sand casting could be addressed by applying squeeze pressure on the sand mold after its development. Pressing is done using a hydraulic press to uniformly squeeze sand into drag and cope sections of the mold, which eventually results in good surface quality and better mechanical properties of the casted product [6].

**Figure 1:**
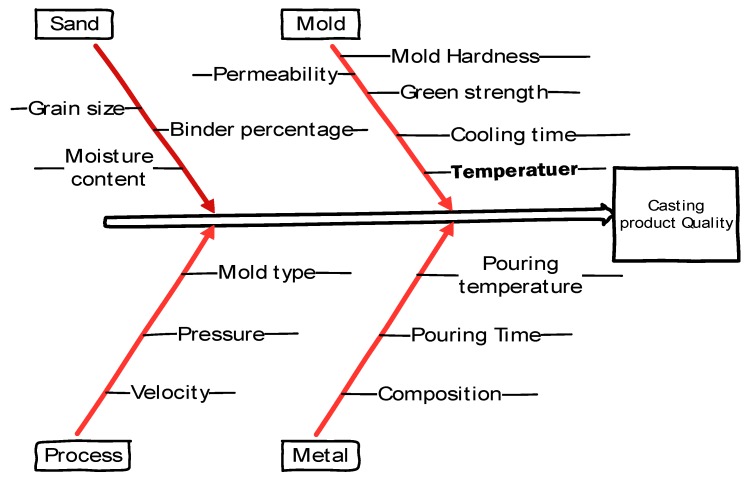
Most influencing causes affecting quality of sand-casted products.

The literature provides evidence on the improvement of casting quality by changing the mold characteristics. Mold characteristics impart a significant impact on mechanical properties. In a study, [7] six sigma methodology was used for improving the casting process. It was claimed that moisture content was found to be the most effective parameter influencing the quality of the casted product. The proposed process reduced the percentage defects greatly along with significant decrement in rework and scrap. In another research, it was deliberated that different molding systems provide variation in cast product properties. Five types of molds were compared, namely CO_2_ mold, cement-bonded, naturally bonded sand mold and metal molds to evaluate their influence on hardness, tensile and impact strength. It was concluded that for cast 6063 Aluminum alloy hardness (HB), tensile strength and ductility provided by the naturally bonded sand mold were the highest among all [8]. Effects of mold parameters, such as one-time recycled sand percentage, bentonite percentage and water percentage were investigated for the behavioral properties of compression strength, permeability, Rockwell hardness and surface quality in other work. The optimal composition of sand mixture were found with one-time recycled sand, bentonite and water percentage. The proposed settings can significantly enhance the mold hardness, compression strength and permeability of the sand mold [9]. Kumar and Singh [10] studied the effects of molding sand composition on the tensile strength of ferrous material. The selected parameters were moisture content, clay content and grain fineness number to examine their role in determining the tensile properties of ferrous alloy using Taguchi experimental design technique. They found that clay was the most contributing parameter while the effect of the remaining parameters was proven to be insignificant for tensile properties of the ferrous alloy. Guharaja S. et al. [11] investigated the effects of moisture contents, green strength, permeability and mold hardness on the quality characteristics of spheroidal graphite (SG) cast Iron. The defects in castings were found to be minimum at optimal values of moisture content, green strength and mold hardness. In another research reported on the same material, it was noticed that the green compression strength has more effect on percent rejection of Si Mo in SG-iron than other factors such as mold hardness, moisture content and permeability [12]. As mentioned earlier, there are many sources that contribute to determining the quality of the casted product; however, these can be categorized as [4,12,13,14,15,16,17] (i) type of sand and sand mixture parameters, (ii) mold parameters, (iii) metal parameters, and (iv) process parameters. The detail of these categories is provided in Figure 1. In other research [18], the Taguchi methodology was used to optimize several parameters, including sand grain size, moisture content, clay content, sprue size, riser size, (diameter to thickness) d/t ratio to improve casting quality. Single and double aluminum blank green sand castings were performed for a robustness comparison considering casting yield strength, surface defects and casting density as response characteristics. The experimental results illustrate that single blank casting is more robust and sensitive to noise factors. Mohammad et al. [19] studied the influence of various parameters, such as mold type, pouring temperature, amount of degasser and holding time on the defect porosity of Al-Si alloy castings. It was observed that the amount of degasser was the most significant parameter for controlling the magnitude of defect porosity. An optimal parametric combination of control variables was also developed using the signal-to-noise ratio method that provides improved casting quality by reducing porosity. Kumar et al. [20] investigated the effects of pouring temperature, grain fineness number, vibration amplitude, time of vibration and degree of vacuum on the surface roughness of evaporative pattern casted product.

It was found that all parameters including grain fineness number and pouring temperature have a significant influence on surface roughness. Shahria et al. [21] evaluated the effect of the percentage composition of sand, bentonite and water on blow holes, pin holes and sand spot of A356 sand-casted alloy. It was narrated that by selecting the optimum combination of sand, bentonite and water, the magnitude of the said defects can be significantly reduced. However, these defects could not be eliminated. A literature summary is also provided in Table 1.

The above literature survey reveals that extensive research work has been carried out in the field of sand casting considering the effect of process parameters and mold-related factors. However, less attention has been paid to formulating the empirical relations of control variables with response characteristics, which is the primary focus of this study. Moreover, the previous research work carried out in the casting of Al A356 was targeted at single response optimization, which is not purposeful for the industry. In this paper, casting of A356 alloy was comprehensively envisaged for formulating the empirical relations of control parameters with surface roughness and mechanical properties. Instead of finding the optimal settings of key control parameters for individual response, multi-objective optimization was carried to simultaneously optimize all the selected responses to augment industrial requirement. Three key sand-casting parameters, namely pouring temperature, pouring velocity and moisture content, were selected as input variables in this paper. The effects of these input parameters were comprehensively discussed using statistical tests and a scanning electron microscopic analysis. Empirical models were successfully developed and validated for ultimate tensile strength, surface roughness and hardness. In addition, optimal parametric combination has also been developed using the desirability approach that ensures the simultaneous optimization of all three responses that have not been done so far.

## 2. Materials and Methods

Aluminum A356 alloy was used as the work piece material in this study. The chemical composition of the material was validated via optical emission spectrometry, the results of which are shown in Table 2. The selected material was casted using the sand-casting technique. The mold for casting the part was prepared via the conventional method. However, the prepared mold was pressed using hydraulic press afterwards to uniformly squeeze sand into the cope and drag sections of the mold. This pressing improves the compaction of the sand into the mold that subsequently provides a compact mold surface. The advantage of that compaction is that it reduces the porosities of the sand mold for better quality of the casted part. The shape of the pattern that was employed during this research was a flat tensile specimen. The shape of the specimen was purposefully selected as the casted product is subjected to tensile test after manufacturing. The total length of the specimen was 165 mm whereas its thickness was 7 mm. Three key parameters, namely pouring temperature, pouring velocity and moisture content, were selected as the control variables in the present research. The rationale for selecting the aforesaid variables as input parameters is their well proven significance with respect to the mechanical and surface properties of the castings [9,17,18]. The control settings of the said parameters were achieved in the following manner. For instance, accurate values of pouring temperature at three different levels were attained using electric muffle furnace which has a maximum temperature range of 1100 °C at 5 KW. Different values of pouring velocities were achieved by varying feeding system according to Bernoulli’s theorem and equation of continuity. For the third parameter (moisture content), Moisture teller was used, which helps in determining the moisture content of the sand. This measurement guides for amendments to ensure that the value of moisture content is at the predefined level. As standard procedure, a sample of 100 grams mixture was placed in a pan and heated up to 110 °C. At this temperature, moisture vaporizes, and the remaining sand is weighed again. Samples moisture content is calculated based on the difference in the weights of pre-heated and post-heated.

**Figure 2:**
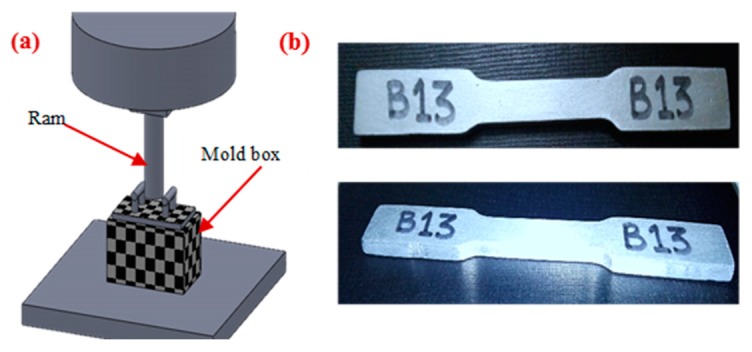
Sand-casting process for Al-alloy; (**a**) Preparation of mold; (**b**) Casted sample.

**Figure 3:**
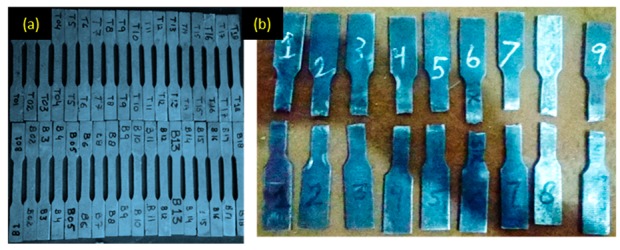
Sand-casting process for Al-alloy: (**a**) Casted samples before tensile testing; (**b**) Selected samples after tensile testing.

The mold used for producing the castings was prepared using green sand (size 47–54 AFS number) mixed with 10% bentonite and 2%–3% coal dust. Afterwards, water was added and thoroughly mixed. Pattern and other gating systems were placed in the drag part and sand was sprinkled in drag through a mesh to get a uniformly packed mold. Then, it was pressed under the hydraulic press shown in Figure 2a. The pressure used for squeezing green sand was 0.80 MPa. The same process was repeated with the cope part and sprue and riser pins were inserted in the cope section. This uniform spray of sand and squeeze gives a high compact ability to the mold. After pouring of melt, it was allowed to solidify for 3–4 min. Afterwards, the mold was shaken out and the casted parts were quenched in water. Water-cooling was carried out for 16 seconds to achieve better mechanical properties. Mechanical properties of squeeze sand-casted products were measured in terms of hardness (HB) and ultimate tensile strength (UTS). For measuring the tensile strength of the cast specimen, a Universal tensile tester (model: 810-MTS) was used, whereas for hardness measurement, a Rockwell hardness testing machine was used. Tensile test specimens were prepared according to ASTM E8/E8M-11 for performing tensile testing on the casted samples, as described in Figure 2b. All the samples that are casted as per the selected DOE are shown in Figure 3a whereas some of the fractured samples are described in Figure 3b. The surface roughness of the quenched specimen was measured using a surface roughness tester (model: SJ-410). The parameters other than the control variables were kept constant and their details are provided in Table 3.

To analyze the effects of different parameters (individually and in combination) on surface roughness, hardness and ultimate tensile strength, the Response Surface Methodology (RSM) technique was used for conducting the experiments. RSM is an effective statistical experimental design methodology to estimate the main, square and interaction effects of the parameters on the selected response attributes. Above all, the developed models of RSM have a high prediction accuracy. In the past, researchers used the Taguchi experimental design technique for modelling the responses. However, the prediction results of those models (developed using Taguchi approach) are less accurate as this approach does not consider the effects of interactions and quadratic terms, whereas in case of the Response surface methodology (RSM), the prediction results are comparatively accurate as this approach also considers the effect of interactions and quadratic terms. Another consideration that limit the use of the Taguchi design is that it can develop only linear models of responses, which is not the case in most of the practical applications. On the other hand, RSM offers the flexibility of developing both quadratic and linear models. Thus, more rigorous models are obtained with the use of the RSM approach. Given the supremacy of the RSM approach, it was employed for the present research. The RSM design technique is commonly in use these day because of its design performance and exemption of cost [4,23,24,25,26]. The ultimate goal of RSM is to examine the region of factor space where operating requirements are satisfied [27,28,29]. The RSM technique was used in the current study to consider pouring temperature, pouring velocity and moisture content as input variables. As mentioned earlier, the selection of these parameters was based on the rationale that the literature illustrates their significant impact on the selected responses. The levels of these parameters were decided based on preliminary trials and the literature survey. The details pertaining to the control parameters and their selected levels are shown in Table 4.

The experiment design was based on a face-centered composite design. The following Equation (1) was used to determine the number of experiments [30]:

n = 2^k^ + 2k + m (1)

where *n* is the number of experiments; *k* is the number of parameters and m is the number of center points. Based on the number of parameters, their levels and center points, it was found that 17 experiments were to be performed as per RSM approach. In the modeled design of experiments, there were 14 factorial points and three center points.

## 3. Results and Discussions

In in this paper, experimentation was performed according to the RSM methodology. In total, 17 experiments were conducted. After completion of each experimental run, the casted sample was removed and subjected to the measurement of mechanical and surface properties. The results of the experimentation are tabulated in Table 5. Experimental results were then thoroughly analyzed using statistical and SEM analyses.

### 3.1. Parametric Significance Analysis

An analysis of variance (ANOVA) was carried out to assess the parametric significance for the selected response attributes [31,32]. A confidence interval of 95% was identified in this study to gauge the significance of the parametric effect. According to this criterion, any control variable having a p-value lower than the defined alpha value, i.e., 0.05 would be considered as significant for the selected response [30,33]. The design matrix developed using the response surface methodology was analyzed using ANOVA. This analysis provides the significance of the model, the influence of process parameters, their significance and the percentage contribution on response measures [34,35]. The results of the analysis are presented in Table 6, Table 7 and Table 8. Based on the ANOVA results shown in Table 6, it can be observed that all three parameters and their squared terms, namely pouring temperature (A) pouring velocity (B) and moisture content, are significant parameters for the hardness of the casted sample. In addition, some interaction terms like pouring temperature and Pouring velocity, pouring temperature and moisture content, were also proven to be significant for this response. It can also be observed that the percentage contribution of the pouring temperature is disproportionately high compared to the rest of the significant control variables. It is pertinent to mention that the value of adjusted R-square is 98.74%, which highlights the adequacy of the proposed model. The ANOVA results pertaining to the ultimate tensile strength (UTS) are provided in Table 7. The results reveal that pouring temperature is the only contributing factor that has a prominent role in governing the tensile strength of the casted specimen. Among the interactions, pouring temperature and moisture content interaction were found to be significant. In addition to these terms, the quadratics of pouring temperature (A^2^) and pouring velocity (B^2^) have also proven to be significant with respect to the tensile strength of the casted specimen. It is worth noting that the developed model for UTS holds an R-square adjusted value higher than 90%, which is proof of the model’s adequacy.

In case of surface roughness, two control variables, pouring temperature and moisture content, were found to be significant. However, the percentage contribution of pouring temperature is the largest one compared to the rest of the significant control variables. Noteworthily, among the interaction terms, only one interaction qualified as significant, i.e., pouring velocity and moisture content. On the other hand, all the squared terms were found to be significant according to the ANOVA results shown in Table 8. The value of R-square adjusted for the surface roughness model is 97%, which proves that the model fits the data fairly well.

### 3.2. 3D Response Surface Plots for Hardness, UTS and SR

**Figure 4:**
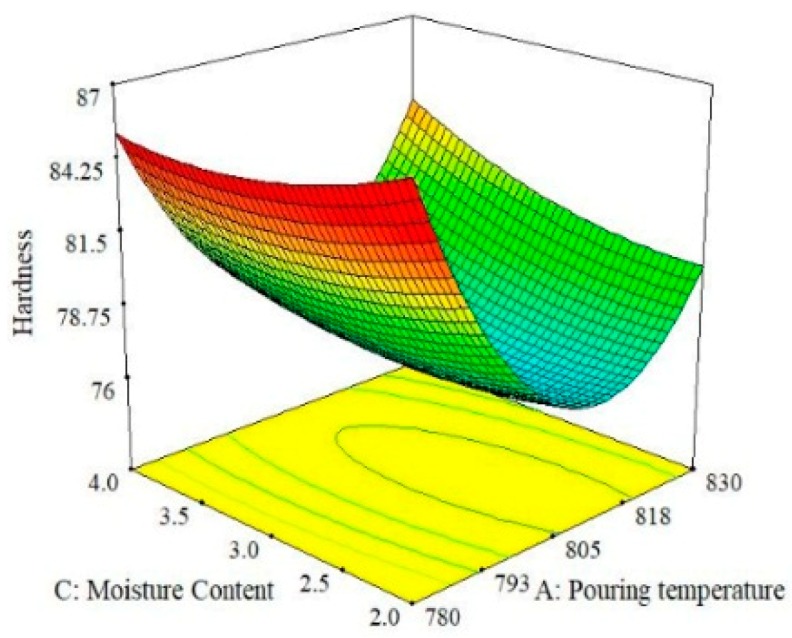
Surface plot for Hardness; M_C_ vs. P_T_.

**Figure 5:**
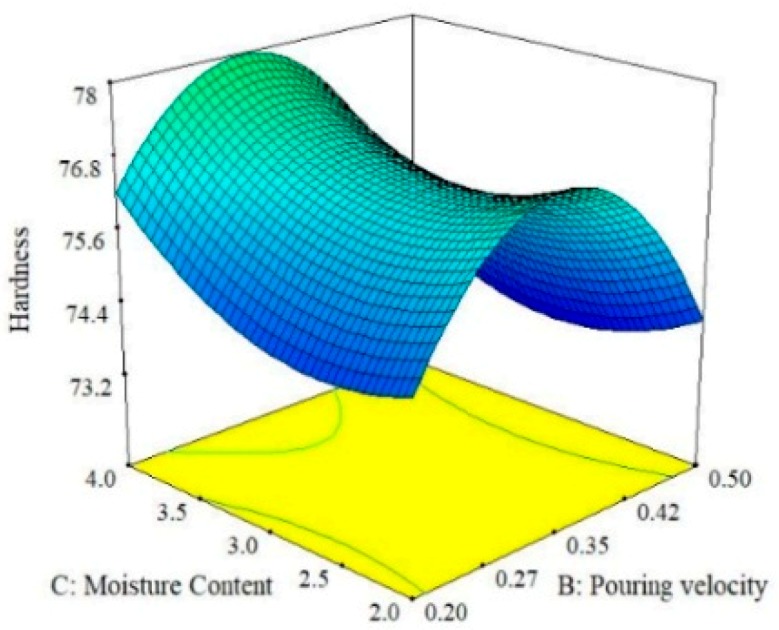
Surface plot for Hardness; M_C_ vs. P_V_.

Three-dimensional response surface plots were used to analyze and graphically visualize the combined effect of the two parameters simultaneously for the selected response [36,37]. These plots primarily highlight the effect of the interaction on the response. For instance, Figure 4 shows the effect of pouring temperature and moisture content on the hardness of the A356 alloy. It can be observed that the pouring temperature is more effect on hardness than moisture content. Moreover, hardness is maximum at the lowest level of pouring temperature because porosity and gas shrinkages defects are minimum at a lower temperature of melt, as reported in previous work [17]. However, a further rise in temperature results in a reduction of hardness, but this pattern persists up to the middle level. Afterwards, once again, hardness tends to increase with the rise in pouring temperature. The value of hardness is observed to be maximum if moisture content is set at its highest level (4%) while keeping the pouring temperature at its lowest level, i.e., 780 °C. Similarly, the effect of pouring velocity and moisture content on the hardness of the squeeze sand-casted alloy is presented in Figure 5. It was found that an increase in hardness occurred when the pouring temperature was increased up to a limit and then started to decrease. Hardness seemed to be more responsive to the change in pouring velocity as compared to moisture content. It can be seen that a middle level of pouring velocity is more effective if a higher hardness value is desired in sand casting. In fact, the pouring velocity directly influences the fluidity of melt in the mold and it has already been reported that hardness increases with the increase in the fluidity of the melt [38,39]. Due to this, the chances of entrapment of gases are reduced, which promotes the formation of a finer microstructure. Eventually, the hardness of the casted sample is increased. Therefore, a careful selection of pouring velocity is essential to ensure the laminar flow of the melt to avoid splashing and to control the fluidity of the melt. Moreover, it has been observed form the surface plots shown in Figure 5 that highest level of moisture content and the middle level of pouring velocity yields a higher hardness of the casted samples.

**Figure 6:**
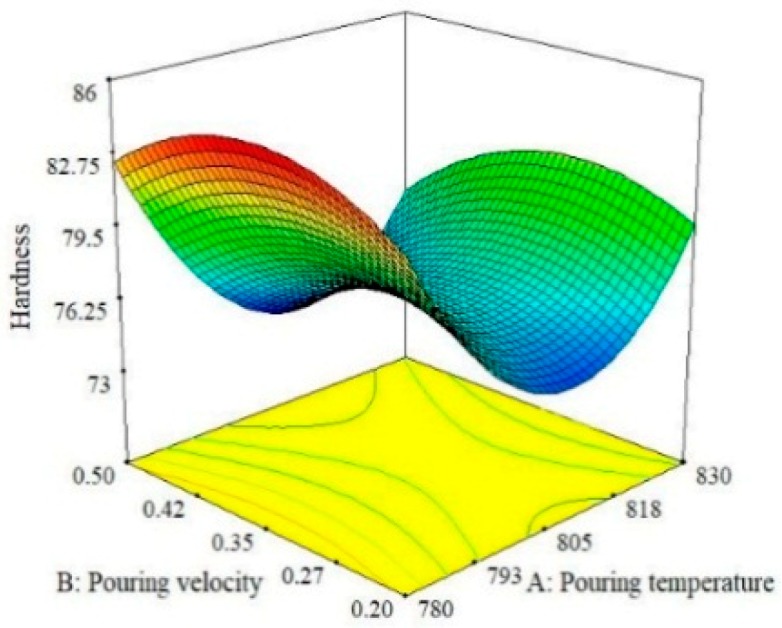
Surface plot for Hardness; P_V_ vs. P_T_.

**Figure 7:**
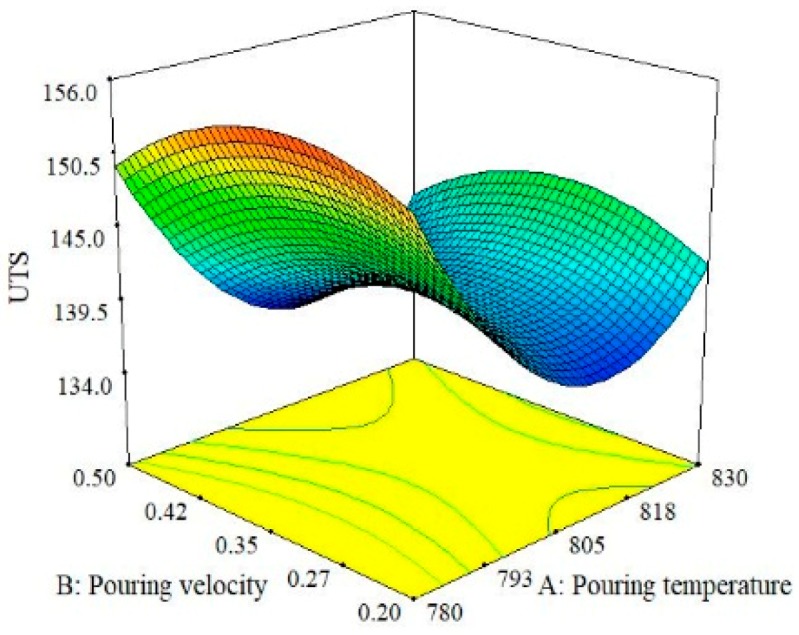
Surface plot for UTS; P_V_ vs. P_T_.

The relationship between the effect of pouring velocity and pouring temperature is illustrated in Figure 6. The results show that hardness is more sensitive to the change in the magnitude of the pouring temperature as compared to pouring velocity. The largest value of hardness can be obtained by selecting the middle value of the pouring velocity and the lower value of the pouring temperature. By observing these three trends for hardness, it can be concluded that hardness is largely more influenced by pouring temperature than pouring velocity and moisture content. This finding agrees with the ANOVA results shown in Table 6. Thus, the optimal combination that guarantees the maximum value of hardness of the casted specimen is level 1 (780 °C) of pouring temperature, level 2 (0.35 m/s) of pouring velocity and level 3(4%) of moisture content as per the 3D-surface plot analysis described in Figure 4, Figure 5 and Figure 6. The effects of the pouring velocity and pouring temperature on the ultimate tensile strength are graphically represented in Figure 7. This 3D plot illustrates that the value of UTS is maximum if the pouring velocity is set at its middle level while the pouring temperature is set at its lowest level. However, in comparison, pouring temperature has a significant influence on ultimate tensile strength, as depicted in Figure 7.

**Figure 8:**
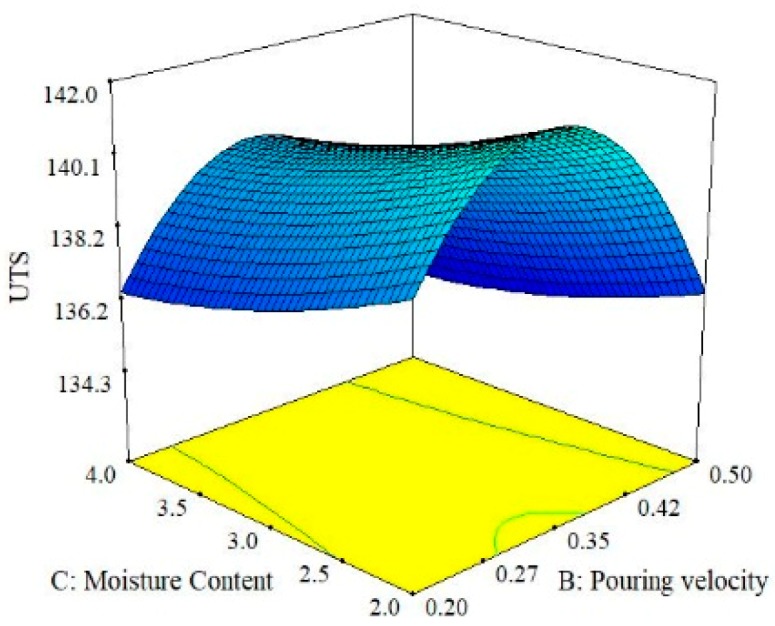
Surface plot for UTS; M_C_ vs. P_V_.

**Figure 9:**
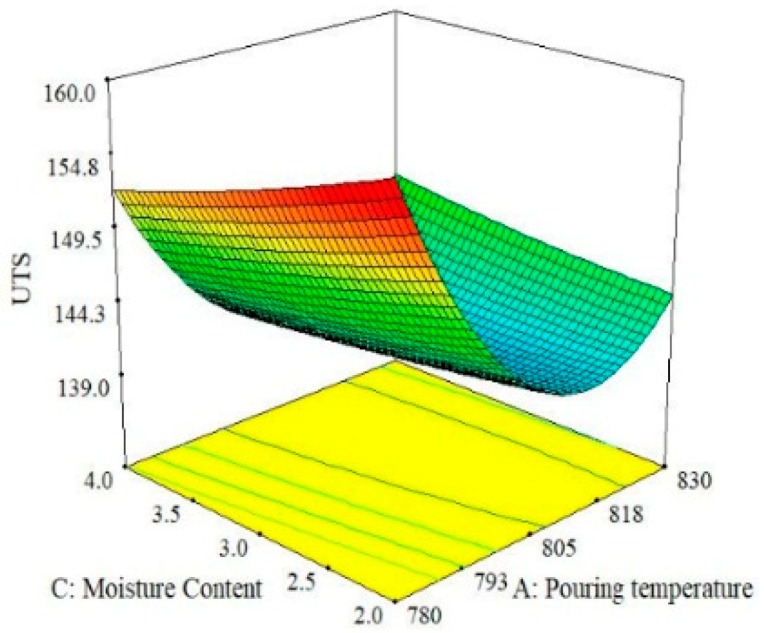
Surface plot for UTS; M_C_ vs. P_T_.

**Figure 10:**
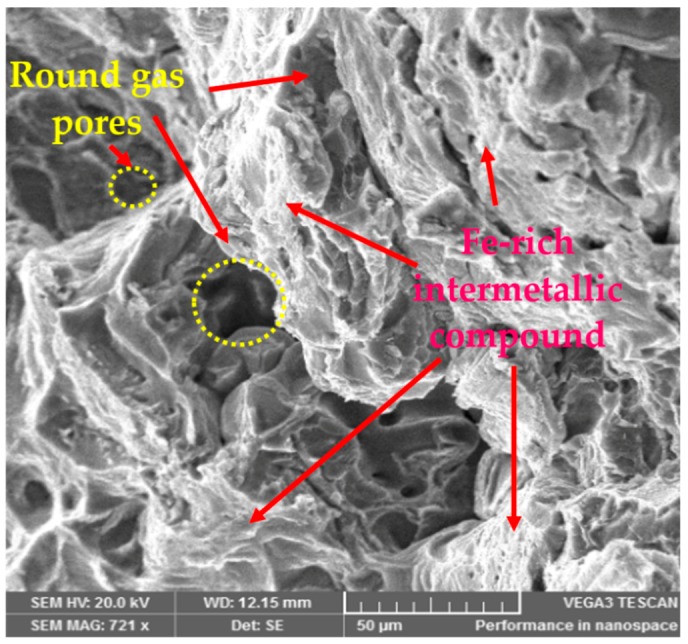
SEM micrograph of fractured casted samples at P_T_ = 830 °C.

When the interaction between moisture content and pouring velocity are plotted against the ultimate tensile strength in Figure 8, it was revealed that pouring velocity heavily influences the UTS as compared to the moisture content. Initially, UTS increases with increase in pouring velocity and decreases with an increase in the moisture content up to certain limit. Moisture contact plays an important role in attaining bonding action for clay with sand; if it increased, then the strength of the mold reduced, which caused casting defects like macro cracks and hot tearing [11,40,41]. However, the maximum value of UTS can be achieved by keeping the pouring velocity at its middle level with the lowest amount of moisture content. The interaction effects of the pouring temperature and the moisture content on the ultimate tensile strength are illustrated in Figure 9. It can be observed that moisture content has a minor effect on UTS as compared to pouring temperature. Moreover, UTS is more responsive to the pouring temperature. The highest value of UTS was obtained at the lowest level of pouring temperature and moisture content. Essentially, the pouring temperature affects the solidification of the melt in the mold. During solidification, it affects the dendrite arm which alters the microstructure of the solidifying melt. The higher pouring temperature enhances the fluidity of the melt, which is likely to provide round gas pores in the solidified structure, as depicted in the SEM micrograph shown in Figure 10. The formation of these pores results in a lower value of UTS. Another reason that contributes to the reduction in UTS magnitude at high P_T_ is the formation of intermetallic compounds with a weak intermetallic bond. These compounds have a flower-like morphology, as highlighted in Figure 10. It has also been reported that these intermetallic compounds have poor deformation properties. Thus, ultimately, the UTS magnitude is compromised. By selecting an adequate level of pouring temperature, better mechanical properties can be achieved [20,42]. In a nut shell, the optimal combination that yields the maximum value of UTS is level 2 (0.35 m/s) of pouring velocity, level 1 (780 °C) of pouring temperature and level 1(2%) of moisture content.

**Figure 11:**
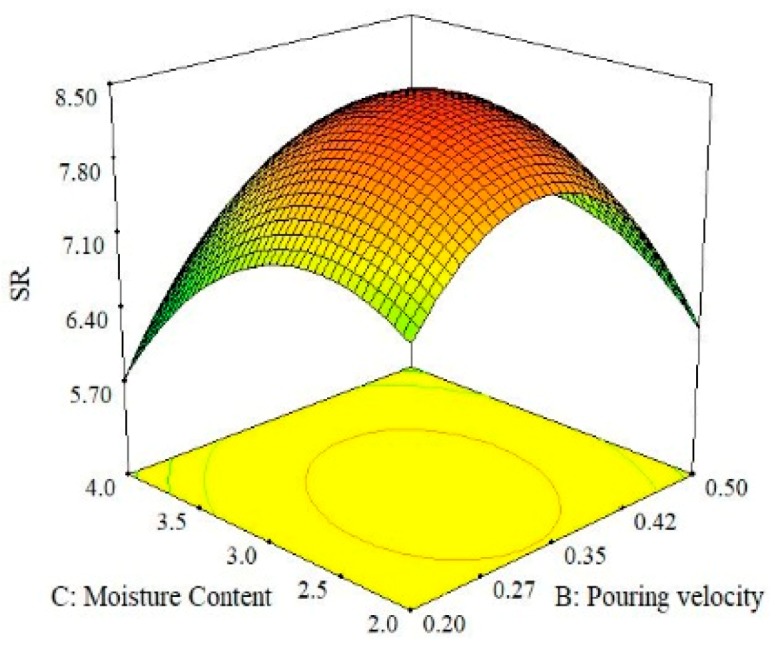
Surface plot for SR; M_C_ vs. P_V_.

**Figure 12:**
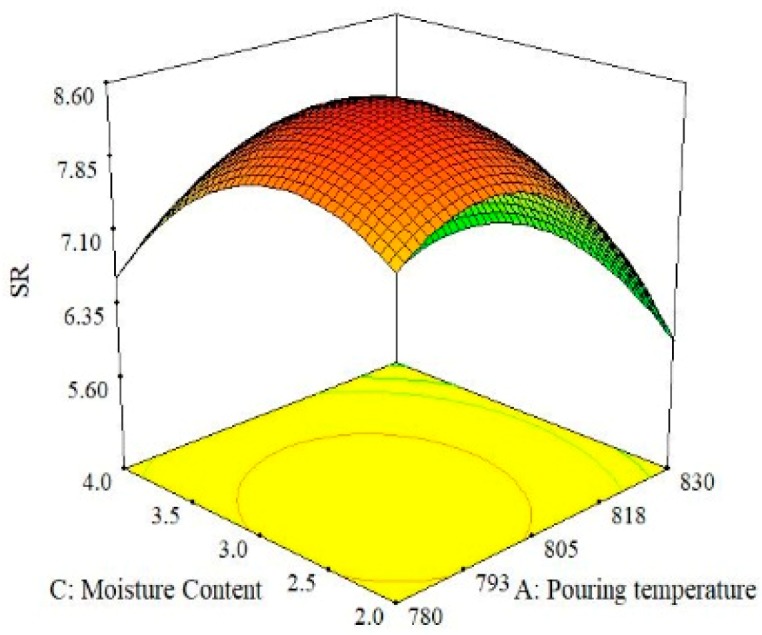
Surface plot for SR; M_C_ vs. P_T_.

**Figure 13:**
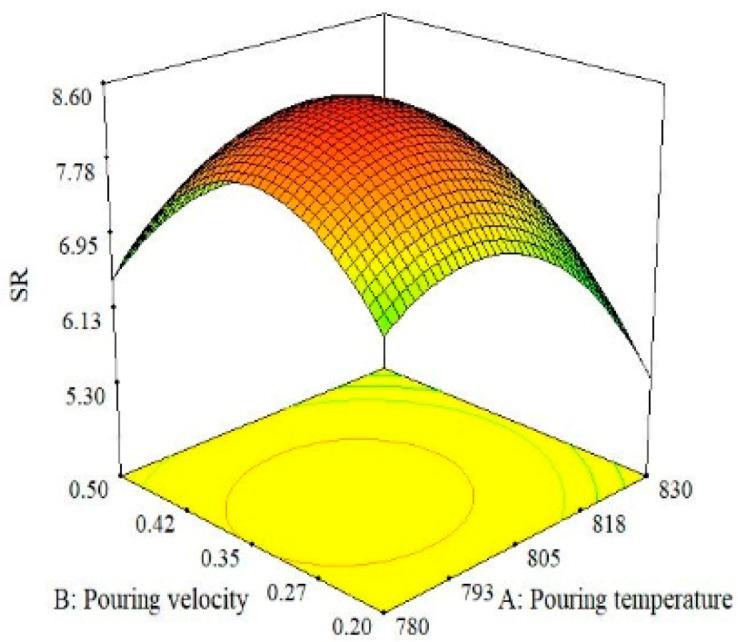
Surface plot for SR; P_V_ vs. P_T_.

The effect of the interaction between moisture content and pouring velocity on surface roughness is illustrated in Figure 11. It was found that the magnitude of surface roughness upsurges with the increase in moisture content and pouring velocity up to a certain point and after that, the threshold surface roughness once again reduces. The best surface quality was obtained at the highest level of moisture content and the lowest level of pouring velocity. Moreover, it was also concluded from the 3D surface plots shown in Figure 11 that middle levels of moisture content and pouring velocity provide the highest surface roughness. When comparing the combined effects of moisture content and pouring temperature on surface roughness in a 3D surface plot (Figure 12), a high value of surface roughness was found at the middle levels of both parameters. A high-quality surface can be achieved by selecting the lowest level of moisture content and the highest level of pouring temperature. A lower moisture content imparts a compact ability to the casted parts and as a result, less defects are produced. Consequently, mechanical properties and surface finish are improved [43,44]. The effects of the pouring velocity and pouring temperature on the surface roughness are illustrated in Figure 13. Both factors significantly influence surface roughness. At middle levels of both pouring velocity and pouring temperature, the surface roughness is at its maximum. The best surface quality can be achieved at the lowest level of pouring velocity and the highest level of the pouring temperature. Based on all the three surface plots provided in Figure 10, Figure 11 and Figure 12, it can be concluded that the optimal parametric combination for surface finish is level 1 (2%) of moisture content, level 1 (0.2 m/s) of pouring velocity and level 3 (830 °C) of the pouring temperature.

### 3.3. Multi-Response Optimization using Desirability Approach

Based on the results obtained in the 3D surface plot analysis described in the previous section, it can be observed that optimal parametric combinations were developed for the selected responses. These optimal combinations can only be used for single response optimization. As for all the three selected response attributes like hardness, UTS and surface roughness, different optimal settings were obtained. Furthermore, the optimal parametric combination that optimizes one response deteriorates the quality of the second response. For instance, in the case of the hardness of the casted sample, it has been noted that level 1(780 °C) of the pouring temperature is optimal but on the other end, for surface roughness, level 3 (830 °C) is optimal. From an industry perspective, all the afore-mentioned responses are important to augment the industrial requirements. Therefore, in the present research, the focus was shifted for simultaneous optimization of responses, including hardness, ultimate tensile strength and surface roughness using the desirability approach. The desirability analysis was carried out on commercial statistical software, i.e., Design Expert (7.0.0 TM). While performing the analysis, all three parameters were set into their ranges and weighted equally. The response measures were set to their optimum requirements and weighted equally to assess fairly. The constraints used for the optimization are presented in Table 9. The optimization was carried out within the selected ranges of the three input parameters. The range of each parameter is provided in Table 4.

According to the number of parameters, their ranges and the considered responses, the software purposes 26 optimal solutions using the desirability approach. The details of these solutions are shown in Table 10. If it is desired to achieve the highest level of hardness at the cost of compromising UTS and SR, the conditions shown in run number 10 should be selected. If the maximum value of UTS is desired by compromising hardness and SR, the conditions shown in run number 12 should be selected. Similarly, if the minimum SR is desired without caring about the value of UTS and hardness, the conditions shown in run number 14 would be a good choice.

**Figure 14:**
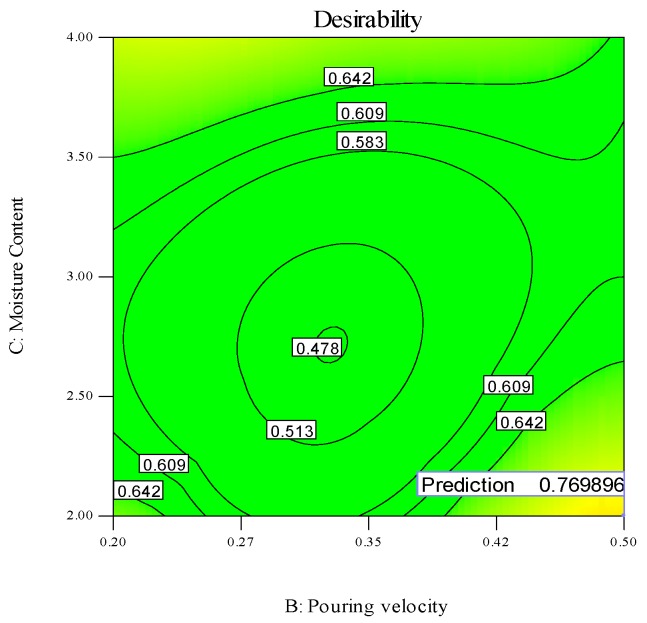
Desirability contour plot, P_V_ vs. M_C_.

**Figure 15:**
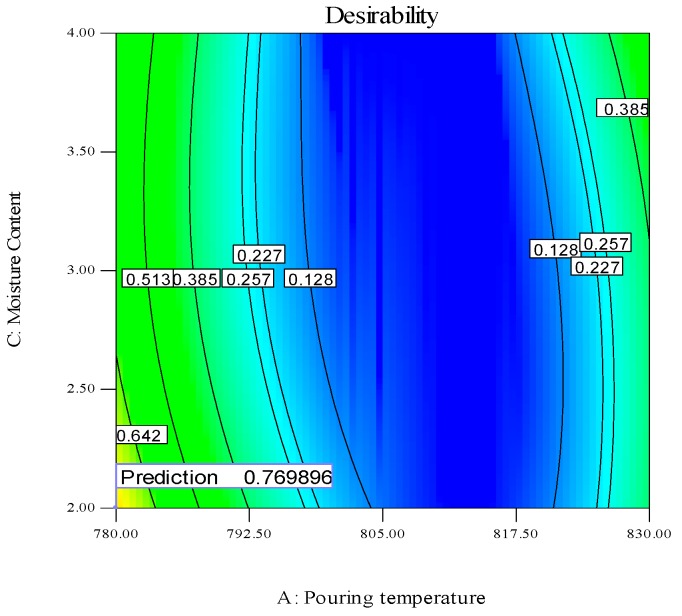
Desirability contour plot, P_T_ vs. M_C_.

**Figure 16:**
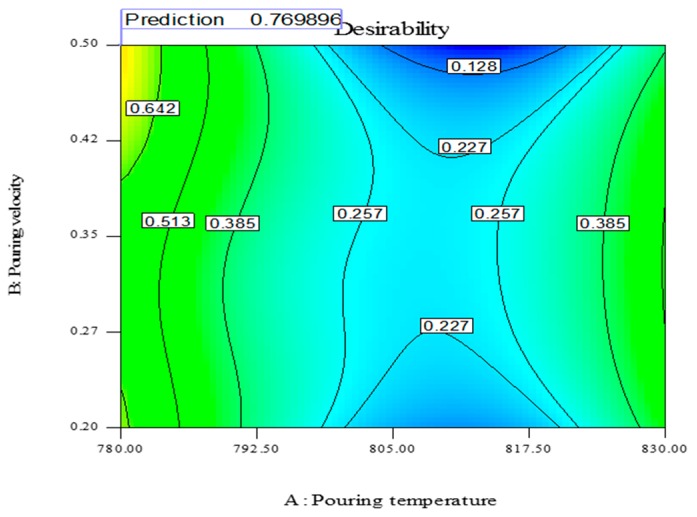
Desirability contour plot, PT vs. PV.

All the aforementioned choices actually depict the possibility of single response optimization using the desirability approach. However, for any optimal value of one response parameter, the overall value of desirability is quite low, as the remaining two responses are compromised, which would not serve the purpose. The optimal setting is required, which ensures the simultaneous optimization of all three responses. The parametric combination which holds the highest value of desirability in the purposed 26 solutions (shown in Table 10) would guarantee the simultaneous optimization of the selected output parameters [45,46]. It has been noted that the parametric combination mentioned in the first row of Table 10 has the maximum value of desirability and was therefore selected for optimizing the hardness, ultimate tensile strength and surface finish of the sand-casted A356 Al-alloy. Hence, the optimal setting that can results in the multi-objective optimization of the selected response characteristics is level 1 (780 °C) of pouring temperature, level 3 (0.5 m/s) of pouring velocity and level 1 (2%) of moisture content. The desirability regarding the selected control variables and output parameters was examined using contour plots. These plots take two input variables at a time against a single response and represent the optimal combination of both variables that can results in maximum values of desirability. These plots are illustrated in Figure 14, Figure 15 and Figure 16.

**Figure 17:**
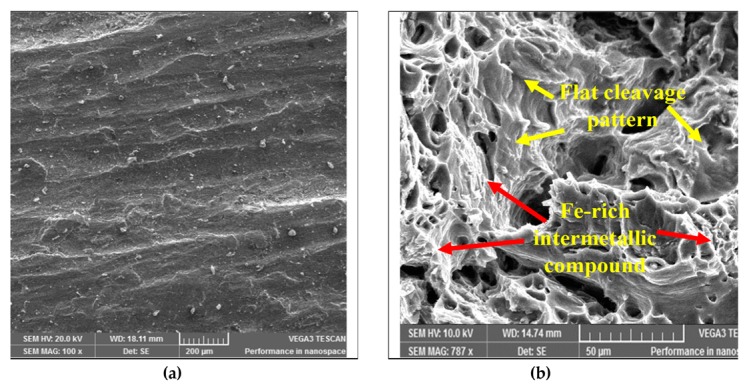
SEM micrographs of the sample casted at optimal settings; (**a**) as casted; (**b**) fractured.

In Figure 14, the optimal moisture content, pouring velocity and maximum desirability are shown at constant value of pouring temperature (780 °C). The point showing the desirability of 0.769896 has coordinates of 2% moisture content, 0.5 m/s pouring velocity and 780 °C pouring temperature. Moving away from this point will result in the decrement of the desirability. The contour plot of the optimal value of moisture content and pouring temperature at a constant pouring velocity of 0.5 m/s and with the maximum desirability is shown in Figure 15. The desirability is maximum (0.769896) at 0.2% moisture content, 0.5 m/s pouring velocity and 780 °C pouring temperature. The magnitude of the desirability of the results becomes lower if we move away from this point. The contour plot of the optimal pouring temperature and pouring velocity at a constant moisture content (2%) is presented graphically in Figure 16. The maximum desirability is achieved at 780 °C pouring temperature, 0.5 m/s pouring velocity and 2% moisture content. It is pertinent to mention that the optimal parametric combination developed via contour plots is the same as that which was developed through the desirability analysis, thus validating the findings of the afore-mentioned analysis. The developed optimal parametric combination was also authenticated via three confirmatory trials. The results of the confirmatory experiments are described in Table 11. It can be noticed that the proposed optimal settings provide the optimal values of responses with reasonable repeatability. The casted sample manufactured at optimal combinations was also examined through an SEM analysis, which confirmed the superiority of the casted specimen, as highlighted in Figure 17.

### 3.4. Mathematical Modelling

**Figure 18:**
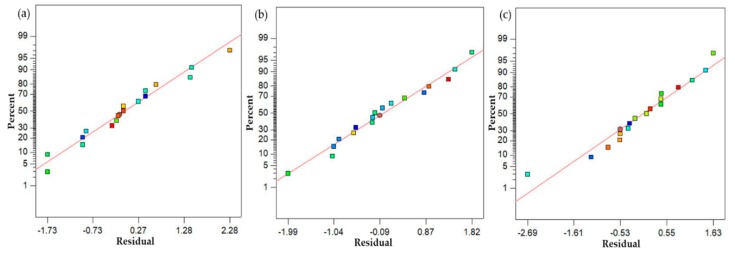
Normal probability plots of residuals; (**a**) hardness; (**b**) UTS; (**c**) surface roughness.

**Figure 19:**
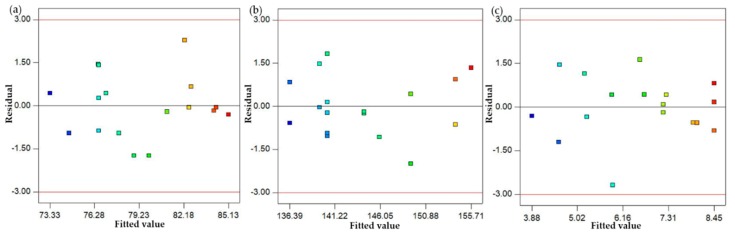
Residual vs. fitted value plots; (**a**) hardness; (**b**) UTS; (**c**) surface roughness.

**Figure 20:**
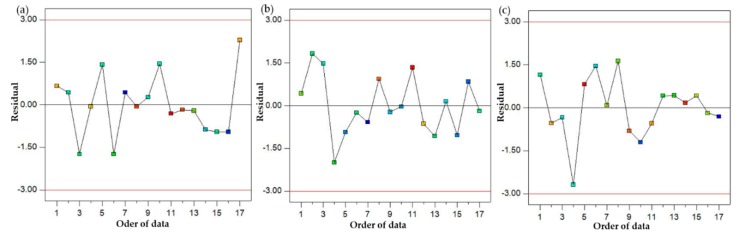
Residual vs. order of data plots; (**a**) hardness; (**b**) UTS; (**c**) surface roughness.

After discussing the parametric effects in detail and developing their optimal parametric combination, mathematical models were formulated in this part of the study. A regression analysis was used to develop the mathematical models for the selected responses. A regression analysis was performed on a commercial statistical software Design Expert (7.0.0 TM) to model the empirical relations of the selected response measures. The obtained empirical models are presented in Equations (2)–(4). The model fit summary suggests that for all the responses, the quadratic model fairly suits the data. The summary of the developed models is illustrated in Table 6, Table 7 and Table 8. Based on the model fit summary, it can be noted that for all the models, the value of adjusted R-square is higher than 90%, which illustrates the high prediction accuracy of the purposed models. In addition, the statistical significance of these models was also checked using ANOVA. A confidence interval of 95% was selected for assessing the statistical significance of the empirical relations. The ANOVA results demonstrate that the p-value of all the mathematical relations was found to be quite lower than the defined alpha value, i.e., 0.05. This means that the purposed empirical models are statistically significant, as illustrated in Table 6, Table 7 and Table 8. The adequacy of the proposed models was also authenticated using the normal probability plot of residuals. The plots of residuals for the three response characteristics, such as hardness, ultimate tensile strength and the surface roughness of the sand-casted A356 al-alloy, are shown in Figure 18. It is clearly presented by the normal probability plots that residuals are normally distributed, which justifies the adequate prediction accuracy of the models. Furthermore, residuals are also plotted against the fitted values and order of data, as presented in Figure 19 and Figure 20. It can be noted that all the residuals are randomly scattered against the fitted values, which is more proof of the accurate prediction of the formulated empirical relations.

Hardness = +7000.479 − (17.043 × P_T_) + (201.365 × P_V_) − (44.440 × M_C_) − (0.146 × P_T_ × P_V_) + (0.05 × P_T_ × M_C_) + (0.0105 × P_T_^2^) − (115.712 × P_V_^2^) + (0.896 × M_C_^2^) (2)

UTS = +140.42 − (4.87 × P_T_) + (2.37 × P_T_ × M_C_) + (10.41 × P_T_^2^) − (4.04 × P_V_^2^) (3)

SR = +8.45 − (0.66 × P_T_) − (0.34 × M_C_) + (0.35 × P_V_ × M_C_) − (1.09 × P_T_^2^) − (1.27 × P_V_^2^) −
(0.86 × M_C_^2^) (4)

#### Model Validation through Confirmatory Tests

**Figure 21:**
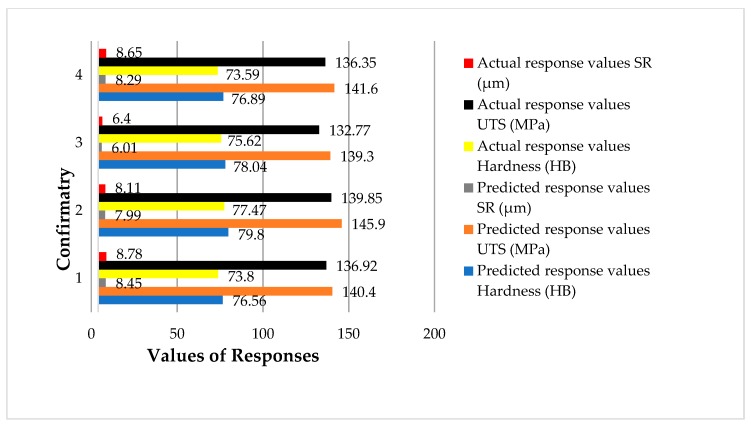
Comparison of the predicted and actual values during confirmatory trials.

Given that the empirical relations were absolutely verified via different statistical methods, the empirical models’ accuracy was also examined through confirmatory trials. Four confirmation experiments were performed by randomly choosing sand casting parametric values. The values of ultimate tensile strength, hardness and surface roughness were measured. The results of the confirmatory trials are provided in Table 12. For each of the confirmatory runs, both experimental and empirical, models’ predicted values were found. Afterwards, the percentage error was calculated using the described relationship as presented in Equation (5) [23,47]. It is worth noting that the formulated empirical relations can accurately predict the values of the selected response attributes with just an average prediction error of 4%. It is well established that if an empirical relation has a prediction error less than 5%, it is considered as accurate for predicting the response characteristic [48,49]. This is also illustrated in Figure 21, which shows the comparison between the predicted and experimental values of the responses during the confirmatory trials.

Percentage error = |(Actual value-Predicted value)/(Predicted value)| × 100
(5)

The ultimate tensile strength of the sand-casted A356 alloy was compared with other alloys, as shown in Table 13. It is clear that the UTS of A36 is higher than that of the others, while surface roughness and hardness also compared with other casting processed alloys, as shown in Table 14.

## 4. Conclusions

This paper investigated the effect of sand casting’s key variables on the mechanical and surface properties of casted A356 Al-alloy. Mold was developed via conventional means and was later subjected to squeezing action by the hydraulic pressure that improves the compaction of the mold. Three significant parameters (pouring temperature, pouring velocity and moisture content) were selected to evaluate their impact on the mechanical and surface properties of sand-casted Al-alloy. Experimentation was performed under RSM-based design of the experiments. The experimental results were analyzed using statistical and SEM techniques to propose the empirical relations of the selected control variables with the responses. Based on the experimental findings and their discussion, following conclusions are drawn:

1. The process of casting was successfully performed for Al-alloy (A356), which provides reasonably good mechanical and surface properties. With respect to mechanical properties, a maximum tensile strength of 157 MPa and hardness of 85 HB were materialized, whereas from a surface roughness perspective, a minimum roughness of 3.8 µm was achieved.
2. ANOVA illustrates that all the selected parameters were proven to be significant with respect to the hardness of the casted samples. However, for UTS, only one parameter was observed to be significant, i.e., pouring temperature. For the case of surface roughness, two control variables, namely pouring temperature and moisture content, were found to be significant as per the ANOVA results. It was also revealed that not only was the influence of the main parametric terms significant, the interaction and quadratic terms also played a noticeable role in determining the magnitude of the response attributes.
3. The experimental results show that the pouring temperature was found to be the significant control variable for all the selected response attributes. Moreover, this is a major contributing parameter for the selected output variables.
4. Based on the results of the 3D surface plots, it was noted that level 2 (0.35 m/s) of pouring velocity, level 1 (780 °C) of pouring temperature and level 1(2%) of moisture content yields the maximum value of UTS, whereas in the case of hardness, level 1 (780 °C) of pouring temperature, level 2 (0.35 m/s) of pouring velocity and level 3 (4%) of moisture content are the optimal levels. In the case of surface roughness, level 1 (2%) of moisture content, level 1 (0.2 m/s) of pouring velocity and level 3 (830 °C) of pouring temperature resulted in the minimum surface roughness.
5. The optimal parametric combination (temperature of 780 °C, pouring velocity of 0.5 m/s and 2% of moisture content) for the simultaneous optimization of all the selected response characteristics was also proposed using the desirability approach. The developed parametric combination has a desirability value of 0.77.
6. Mathematical models were developed for all three responses. The prediction accuracy of these models was also validated via confirmatory tests. The results of the confirmatory trials demonstrate that the proposed models hold a fairly high prediction accuracy. There exits only a 4% prediction error on average. The models’ significance and validity were also statistically witnessed.

## Figures and Tables

**Table 1:** Literature summary.

Serial. No	Material	Process Parameters	Methodology	Responses	Findings	Ref.
1	6063 Al alloy	Mold types: CO_2_ process, cement-bonded, metallic, naturally bonded sand	Simple graphical method	Hardness, Tensile and impact strength	Higher hardness and tensile strength obtained in case of naturally bonded sand mold, while maximum impact strength observed for metallic mold.	[8]
2	Iron	Recycled sand (%), bentonite (%), M_C_ (%)	RSM	Green compression strength (GCS), permeability	Maximum GCS (53,090 N/m^2^) and permeability (30) obtained at 93.3% one-time recycled molding sand, 1.7% M_C_ and 5% bentonite.	[9]
3	FG200 ferrous metal	M_C_ (%), clay (%), grain fineness no. (GFN)	Taguchi method (L9 array)	Tensile strength	Tensile significantly affected by clay%, maximum tensile strength (197 MPa) attained by selecting clay (8.3%), GFN (80) and M_C_ (3.7%).	[10]
4	SG cast iron	M_C_ (%), GCS (g/cm^2^), permeability no., mold hardness (M_H_)	Taguchi method (L18 array)	Casting defects %	Minimum casting defects observed at optimal values of M_C_ (2.6%), GCS (950 g/cm^2^), and permeability no. (235) and M_H_ (80).	[11]
5	(SiMo) SG iron	M_H_, M_C_ (%), permeability no., GCS (g/cm^2^)	Taguchi method (L18 array)	Casting defects %	Better quality of castings attained at optimal values of mould hardness no. (90), permeability no (135), GCS (1400 gm/cm^2^), and M_C_ (4.75%).	[12]
6	Cast iron	M_C_ (%), permeability, vent holes (Nos), GCS (kg/cm^2^), loss of ignition (%), poring time T_P_ (s), volatile (%), pouring temperature P_T_ (°C), mold pressure M_P_ (kg/cm^2^)	Taguchi method (L27 array)	Casting defects %	Minimum casting defects % observed at optimal parameters values: permeability (120), GCS (1 kg/cm^2^), vent holes (Nos-10), loss of ignition (3.5%), volatile (2.1%), M_C_ (3.6%), M_P_ (5 kg/cm^2^), T_P_ (5s), P_T_ (1400 °C).	[13]
7	Aluminium alloy	Binder types: Clay (%), molasses (%), oil (%)	CFD simulation	Cooling rate	Clay was found best binder.	[16]
8	Aluminium 319 alloy	P_T_ (750 °C)	3D sand printing, computational simulation	Mold filling velocity	3DSP is better geometrical choice for complex gating systems required to diminish turbulence.	[17]
9	Aluminium alloy	Metal flow rate, P_T_ (°C), humidity	Taguchi method	Casting yield, density, surface defects	Single blank Al casing is more robust than double blank Al sand casting, metal flow rate and P_T_ significantly effects on the responses.	[18]
10	Al-Si (A356) alloy	Type of sand mold: (Sodium-silicate, dry, air set), P_T_ (°C), Degasser (%), holding time (s)	Taguchi method	Porosity %	P_T_ significantly influence at the casting quality.	[19]
11	Al-7% Si alloy	Degree of vacuum (mmHg), P_T_ (°C), GFN, amplitude (μm) and time of vibration (s)	RSM	Surface roughness	High P_T_ reduce the surface tension melt and facilitates the sucking of melt into capillaries developed among the sand grains as the result SR enhanced.	[20]
12	A356 Al alloy	Sand (%), M_C_ (%), bentonite (%)	Simple graphical method, (ANOVA)	Casting defects	Castings have less defects at best combination of 90% sand, 5% bentonite, and 5% M_C_.	[21]
13	A356 Al alloy	87% silica sand, 3% M_C_, and 10% bentonite, P_T_ 720 °C, cooling rate, degassing time	Simple graphical method	Ultimate tensile strength	26 °C/min cooling rate gives better tensile strength of casting due to lower porosity% and secondary arm spacing.	[22]
14	Al-3.5% Cu alloy	P_T_ (°C), pressure (MPa)	RSM	Ultimate tensile strength, hardness, % elongation	P_T_ prominently affecting the mechanical properties of casted product.	[23]

**Table 2:** Chemical composition of A356 alloy.

Elements	Si	Mg	Mn	Sn	Fe	Al
weight %	7.32	0.365	0.25	0.031	0.269	balance

**Table 3:** Constant parameters with their ranges.

Parameters	Value	Parameters	Value
Sand grain size	AFS 50	Environment temperature	26 °C
Pouring time	10 s	Pouring height	7 cm
Binder ratio	85–15 wt %	Squeeze Pressure	0.8 MPa

**Table 4:** Process parameters and their levels.

Parameters	Units	Low	Medium	High
Pouring temperature (P_T_)	°C	730	780	830
Pouring velocity (P_V_)	m/s	0.2	0.35	0.5
Moisture Content (M_C_)	%	2	3	4

**Table 5:** Design matrix with output responses.

Run	P_T_	P_V_	M_C_	Hardness	UTS	SR	Run	P_T_	P_V_	M_C_	Hardness	UTS	SR
	(°C)	(m/s)	(%)	(HB)	(MPa)	(µm)		(°C)	(m/s)	(%)	(HB)	(MPa)	(µm)
1	780	0.20	4	82.9	150.0	5.50	**10**	830	0.50	2	77.0	139.5	4.25
2	805	0.35	2	77.2	143.8	7.80	**11**	780	0.35	3	85.0	157.8	7.90
3	830	0.20	2	78.3	142.0	5.17	**12**	780	0.50	2	84.1	153.0	5.99
4	780	0.50	4	82.5	146.0	5.23	**13**	830	0.35	3	81.0	144.3	6.80
5	805	0.35	3	77.3	138.7	8.70	**14**	805	0.35	3	76.1	140.7	8.50
6	830	0.50	4	79.3	143.9	4.95	**15**	805	0.35	4	77.5	138.5	7.35
7	805	0.50	3	73.5	135.4	7.20	**16**	805	0.20	3	74.2	137.8	7.13
8	780	0.20	2	84.3	155.6	7.00	**17**	830	0.20	4	83.0	144.0	3.80
9	805	0.35	3	76.7	140.0	8.20	-	-	-	-	-	-	-

**Table 6:** ANOVA for hardness.

Source	Sum of Square	Df	Mean Squares	F Value	*p*-Value	-
Model	212.01	8	26.50	78.67	<0.0001	significant
A-Pouring temperature	40.80	1	40.80	121.12	<0.0001	-
B-Pouring velocity	3.97	1	3.97	11.78	0.0089	-
C-Moisture Content	1.85	1	1.85	5.49	0.0472	-
AB	2.42	1	2.42	7.18	0.0279	-
AC	12.50	1	12.50	37.11	0.0003	-
A^2^	114.82	1	114.82	340.84	<0.0001	-
B^2^	18.16	1	18.16	53.91	<0.0001	-
C^2^	2.15	1	2.15	6.39	0.0354	-
Residual	2.70	8	0.34	-	-	-
Lack of Fit	1.98	6	0.33	0.91	0.6064	not significant
Pure Error	0.72	2	0.36	-	-	-
Cor Total	214.71	16	-	-	-	-
Std. Dev.		0.58	R^2^		0.9874	-
Mean		79.41	Adj R^2^		0.9749	-
C.V. %		0.73	Pred R^2^		0.9215	-
PRESS		16.86	Adeq Precision		27.941	-

**Table 7:** ANOVA for UTS.

Source	Sum of Square	Df	Mean Squares	F Value	*p*-Value	-
Model	617.93	4	154.48	37.41	<0.0001	significant
A-Pouring temperature	237.17	1	237.17	57.43	<0.0001	-
AC	45.13	1	45.13	10.93	0.0063	-
A^2^	328.46	1	328.46	79.53	<0.0001	-
B^2^	49.33	1	49.33	11.94	0.0048	-
Residual	49.56	12	4.13	-	-	-
Lack of Fit	47.50	10	4.75	4.61	0.1913	not significant
Pure Error	2.06	2	1.03	-	-	-
Cor Total	667.49	16	-	-	-	-
Std. Dev.		2.03	R^2^		0.9258	-
Mean		144.18	Adj R^2^		0.9010	-
C.V. %		1.41	Pred R^2^		0.8476	-
PRESS		101.70	Adeq Precision		17.529	-

**Table 8:** ANOVA for SR.

Source	Sum of Square	Df	Mean Squares	F Value	*p*-Value	-
Model	35.51	6	5.92	51.19	< 0.0001	significant
A-Pouring temperature	4.42	1	4.42	38.25	0.0001	-
C-Moisture Content	1.14	1	1.14	9.88	0.0104	-
BC	0.99	1	0.99	8.54	0.0153	-
A^2^	3.16	1	3.16	27.29	0.0004	-
B^2^	4.32	1	4.32	37.39	0.0001	-
C^2^	1.98	1	1.98	17.15	0.0020	-
Residual	1.16	10	0.12	-	-	-
Lack of Fit	1.03	8	0.13	2.03	0.3713	not significant
Pure Error	0.13	2	0.06	-	-	-
Cor Total	36.67	16	-	-	-	-
Std. Dev.		0.34	R^2^		0.9685	-
Mean		6.56	Adj R^2^		0.9496	-
C.V. %		5.19	Pred R^2^		0.9024	-
PRESS		3.58	Adeq. Precision		20.945	-

**Table 9:** Constraints for optimization.

Name	Goal	Lower Limit	Upper Limit	Lower Weight	Upper Weight	Importance
Pouring temperature	within range	780	830	1	1	3
Pouring velocity	within range	0.2	0.5	1	1	3
Moisture Content	within range	2	4	1	1	3
Hardness	maximize	73.5	85	1	1	3
UTS	maximize	135.4	157.8	1	1	3
SR	minimize	3.8	8.7	1	1	3

**Table 10:** Optimized conditions of the process parameters for optimal output responses.

No.	P_T_ (°C)	P_V_ (m/s)	M_C_ (%)	Hardness	UTS	SR	Desirability	
1	780	0.5	2	84.48	153.46	5.80	0.77	Selected
2	780.2	0.5	2	84.56	153.61	5.84	0.762	
3	780.32	0.5	2	84.27	153.12	5.82	0.758	
4	780	0.5	2.08	84.37	153.37	5.98	0.749	
5	780	0.48	2.06	85.00	154.63	6.32	0.747	
6	780	0.23	4	83.77	150.81	5.72	0.72	
7	780	0.22	4	83.73	150.75	5.70	0.72	
8	780	0.22	4	83.63	150.62	5.64	0.72	
9	780.03	0.23	4	83.80	150.83	5.74	0.719	
10	780	0.31	4	85.10	152.46	6.50	0.699	
11	780	0.24	3.89	83.96	151.53	6.15	0.699	
12	781.37	0.45	2	85.00	155.02	6.84	0.693	
13	830	0.27	4	83.70	146.40	5.07	0.686	
14	830	0.26	4	83.66	146.30	5.02	0.686	
15	780	0.39	4	84.89	151.62	6.55	0.68	
16	830	0.31	4	83.88	147.24	5.50	0.678	
17	829.71	0.27	4	83.55	146.18	5.09	0.676	
18	780	0.41	4	84.54	150.92	6.42	0.676	
19	830	0.33	4	83.84	147.35	5.59	0.673	
20	780	0.44	4	84.01	149.92	6.21	0.67	
21	780	0.42	3.97	84.44	150.86	6.46	0.67	
22	780.21	0.42	4	84.32	150.54	6.40	0.669	
23	830	0.34	4	83.75	147.39	5.67	0.666	
24	830	0.38	4	83.21	147.06	5.73	0.643	
25	830	0.42	4	82.33	146.15	5.62	0.614	
26	830	0.33	2	80.37	144.94	5.99	0.52	

**Table 11:** Results of the confirmatory experiments.

Sr. No.	Process Parameters			Responses		
	P_T_ (°C)	P_V_ (m/s)	M_C_ (%)	Hardness (HB)	UTS (MPa)	SR (µm)
1	780	0.5	2	83.94	152.15	5.71
2	780	0.5	2	83.26	151.59	5.69
3	780	0.5	2	82.15	151.83	5.75
Average experimental values				83.12	151.86	5.72
Standard deviation				**0.84**	**0.26**	**0.03**
Predicted value				84.48	153.46	5.80
Error (%)				***1.61***	***1.04***	***1.44***

**Table 12:** Confirmatory tests with actual and predicted responses with percentage error.

Run	Process Parameters			Predicted Response Values			Actual Response Values			Percentage Error		
	PT (°C)	PV (m/s)	MC (%)	Hardness (HB)	UTS (MPa)	SR (µm)	Hardness (HB)	UTS (MPa)	SR (µm)	Hardness (HB)	UTS (MPa)	SR (µm)
1	805	0.35	3	76.56	140.40	8.45	73.80	136.92	8.78	3.60	2.48	3.91
2	790	0.4	3.5	79.80	145.90	7.99	77.47	139.85	8.11	2.92	4.33	1.50
3	815	0.25	4	78.04	139.30	6.01	75.62	132.77	6.40	3.10	4.68	6.48
4	800	0.4	2.5	76.89	141.60	8.29	73.59	136.35	8.65	4.29	3.70	4.34

**Table 13:** Comparison of the UTS of the current work with others.

Comparison		UTS (MPa)	% Improvement	Reference
A356 alloy (Maximum UTS achieved in current study: 157.8 MPa)	6061 aluminium alloy	105.9	49%	[22]
	A356	148.0	6.6%	[50]

**Table 14:** Comparison of SR and hardness of current work with others.

Material, Process	Minimum SR	Maximum SR	Hardness	Reference
A356 (sand casting, present work)	3.80	8.70	85.0	
A356 (Low foam casting-under gravity)	6.30	12.50	79.8	[51,52]
% improvement	65.79%	43.68%	6.52%	
A356 (Low foam casting- with vacuum and low pressure)	6.30	12.50	80.9	[51,52]
% improvement	65.79%	43.68%	5.07%	
A713 (sand casting)	-	-	68.5	[53]
% improvement	-	-	23.93%	
